# S-allyl Cysteine, a Garlic Compound, Produces an Antidepressant-Like Effect and Exhibits Antioxidant Properties in Mice

**DOI:** 10.3390/brainsci10090592

**Published:** 2020-08-26

**Authors:** Elizabeth Ruiz-Sánchez, José Pedraza-Chaverri, Omar N. Medina-Campos, Perla D. Maldonado, Patricia Rojas

**Affiliations:** 1 Laboratory of Neurotoxicology, National Institute of Neurology and Neurosurgery “Manuel Velasco Suárez”, SS, Av. Insurgentes Sur No. 3877, Mexico City 14269, Mexico; elizabeth.ruiz@innn.edu.mx; 2 Facultad de Química, Departamento de Biología, Universidad Nacional Autónoma de México, CDMX 04510, Mexico; pedraza@unam.mx (J.P.-C.); omarnoelmedina@gmail.com (O.N.M.-C.); 3 Laboratory of Cerebral Vascular Pathology, National Institute of Neurology and Neurosurgery “Manuel Velasco Suárez”, SS, Av. Insurgentes Sur No. 3877, Mexico City 14269, Mexico; maldonado.perla@gmail.com

**Keywords:** S-allyl cysteine, Porsolt forced swim test, antidepressant-like effect, antioxidant defense, antioxidant enzymes, oxidative stress

## Abstract

Depression is a psychiatric disorder, and oxidative stress is a significant mechanism of damage in this mood disorder. It is characterized by an enhancement of oxidative stress markers and low concentrations of endogenous antioxidants, or antioxidants enzymes. This suggests that antioxidants could have an antidepressant effect. S-allyl cysteine (SAC) is a compound with antioxidant action or free radical scavenger capacity. The purpose of the current research was to evaluate the antidepressant-like effect as well as the antioxidant role of SAC on a preclinical test, using the Porsolt forced swim test (FST). SAC (30, 70, 120, or 250 mg/kg, ip) was administered to male BALB/c mice daily for 17 days, followed by the FST at day 18. Oxidative stress markers (reactive oxygen species, superoxide production, lipid peroxidation, and antioxidant enzymes activities) were analyzed in the midbrain, prefrontal cortex, and hippocampus. SAC (120 mg/kg) attenuated the immobility scores (44%) in the FST, and protection was unrelated to changes in locomotor activity. This antidepressant-like effect was related to decreased oxidative stress, as indicated by lipid peroxidation and manganese-superoxide dismutase (Mn-SOD) activity in the hippocampus. SAC exerts an antidepressant-like effect that correlated, in part, with preventing oxidative damage in hippocampus.

## 1. Introduction

Depression is a prevalent and debilitating mental illness. It is related to a severe negative impact on the quality of life, and an increment in morbidity/mortality. It is identified by diverse symptoms related to impaired behavior, and affective, cognitive, and somatic functioning. It affects up to 20% of the population worldwide and can lead to early death, with social and economic consequences [1]. Although the molecular mechanisms underlying depression have not been elucidated, several lines of evidence indicate that oxidative stress, due to an overproduction of free radicals and/or decreased antioxidant capacity, plays a substantial part in this pathology [2,3].

Depressed patients have been described as having low concentrations of endogenous antioxidant compounds (coenzyme Q10, vitamin E, and zinc) and antioxidant enzymes, for example glutathione peroxidase (GPx), along with oxidative damage to deoxyribonucleic acid (DNA), alterations in mitochondrial function, and enhancement of lipid peroxidation (LPx) products [4,5]. These studies suggest that depression is associated with modifications in the balance between pro- and anti-oxidative processes.

In particular, the brain is susceptible when excessive reactive oxygen species (ROS) surpass the capacity of enzymatic and non-enzymatic antioxidants such as manganese superoxide dismutase (Mn-SOD), and copper-zinc SOD (Cu,Zn-SOD), catalase, GPx, glutathione reductase (GR), vitamin E, lipoic acid, and ferritin, among others. This results in oxidative stress, and in cell disturbance that leads to damage to lipids, proteins, and DNA, with subsequent structural and functional damage to cell membranes, inactivation of enzymes, and cell death [6].

Therefore, it is justifiable to suggest that several compounds with antioxidant action may be efficient in treating depression, since classical antidepressants have inconsistent efficacy, and many of them produce severe unwanted side effects. Indeed, it has been shown that antioxidants such as *Ginkgo biloba* extract (EGb 761), curcumin, and resveratrol can exert an antidepressant-like action accompanied by an antioxidant effect against oxidative stress [7,8,9,10].

Garlic (*Allium sativum*) is rich in sulfur-containing compounds, including S-allyl cysteine (SAC) and diallyl disulfide [11]. SAC is the active compound and the most abundant organosulfur molecule found in aged garlic extract and has multiple beneficial effects such as anti-cancer action [12], cholesterol-lowering action [13], and antioxidant and ROS scavenging effects [14]. SAC is known to scavenge ROS as superoxide anion (O_2_^•−^), hydrogen peroxide (H_2_O_2_), hydroxyl radical (•OH), and peroxynitrite anion (ONOO^−^) [14]. It is a potent antioxidant agent, less toxic than other garlic antioxidants, water-soluble, stable, and easily absorbed by diverse tissues, including the brain [14].

SAC also has neuroprotective effects against ischemia-induced oxidative damage in rat brains [14], together with a decrease in oxidative stress markers in animal models of neurodegeneration such as Parkinson’s [15] and Alzheimer´s diseases [16]. The Porsolt forced swim test (FST) is the most common and established preclinical test for evaluating antidepressant-like responses under the stress induced by forcing the mice to swim [17], with good reliability and predictive validity [18]. Moreover, oxidative damage has been described in this model [8,19,20]. Thus, the present study aimed to investigate whether SAC exerts an antidepressant-like response associated with its antioxidant properties against FST-induced oxidative stress. We hypothesized that SAC administration will reduce depressive-like behavior, and this could be directly associated, in part, with its antioxidant action. Therefore, we analyzed immobility scores in the FST, spontaneous locomotor activity, LPx, ROS levels, O_2_^•−^ production, and Mn-SOD, Cu,Zn-SOD, GPx, and GR activities.

## 2. Materials and Methods

### 2.1. Drugs and Reagents

SAC was synthetized as described previously [21], and was compared with a SAC standard based on melting point, ^1^H nuclear magnetic resonance spectroscopy, and infrared spectroscopy [21]. GR, NADPH, xanthine, xanthine oxidase, nitroblue tetrazolium (NBT), bovine serum albumin (BSA), ethylenediaminetetraacetic acid (EDTA), reduced glutathione (GSH), glutathione disulfide (GSSG), H_2_O_2_, sodium diethyldithyocarbamate trihydrate (DDC), trichloroacetic acid (TCA), dihydroethidium (DHE), 1,1,3,3-tetramethoxypropane, and imipramine-HCl were from Sigma-Aldrich (St. Louis, MO, USA). Thiobarbituric acid (TBA), 2′,7′-dichlorofluorescein diacetate (DCFH-DA), and 2′,7′-dichlorofluorescein (DCF) were from Merck (Darmstadt, Germany). All other reagent-grade chemicals were commercially available.

### 2.2. Animals

A total of 178 male BALB/c mice (Harlan, Mexico City, Mexico) at 11 to 13 weeks of age (25–30 g) were used in this experimental protocol. The animals were housed five per cage and maintained under a 12 h light–dark cycle (lights on at 7:00 a.m.), a relative humidity of 40% at 21 ± 2 °C, with water and food ad libitum up to the time of the experiment. The experimental protocol was carried out following the Mexican regulations for the Care and Use of Laboratory Animals (NOM-062-ZOO-1999), and it was approved by the Animal Care and Use Committee at the National Institute of Neurology and Neurosurgery. We made all efforts to minimize the number of animals used and their suffering. Experimental procedures were conducted between 9:00 and 15:00 h in the light phase of the circadian cycle by one experimenter. We allow a habituation time of 1 h before the experimental procedures.

### 2.3. Experimental Design

**Figure 1:**
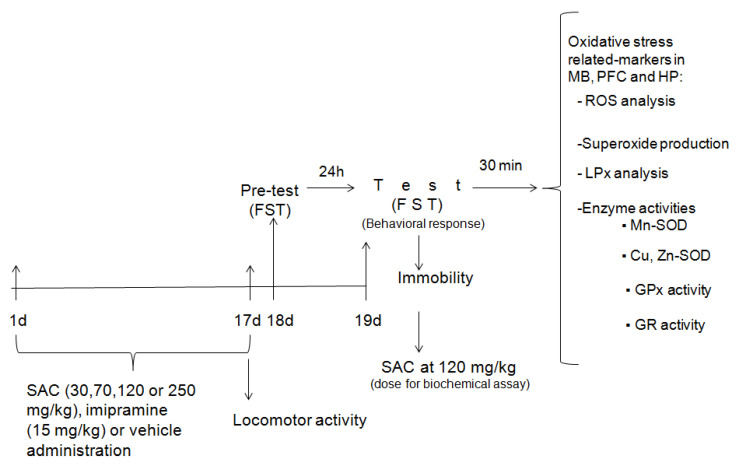
Schematic experimental protocol. FST = forced swimming test; SAC = S-allyl cysteine; MB = midbrain; PFC = prefrontal cortex; HP = hippocampus; ROS = reactive oxygen species; LPx = lipid peroxidation; Mn-SOD = manganese-superoxide dismutase; Cu,Zn-SOD = copper, zinc-superoxide dismutase; GPx = glutathione peroxidase; GR = glutathione reductase.

Experimental animals were divided in seven treatment groups: Group I: vehicle-treated + non-FST; Group II: vehicle-treated + FST; Group III: imipramine + FST; Group IV: SAC (30 mg/kg) + FST; Group V: SAC (70 mg/kg) + FST; Group VI: SAC (120 mg/kg) + FST; and Group VII: SAC (250 mg/kg) + FST. The control groups (I and II) were administered with saline solution. A classical antidepressant drug, imipramine, was used as a positive control. The experimental protocol of the current study is represented in Figure 1.

The antidepressant-like effect of SAC was evaluated by administering different doses (30, 70, 120, or 250 mg/kg, ip; *n* = 11–15 animals per group), imipramine (15 mg/kg, ip; *n* = 13), or vehicle (ip; *n* = 10) daily for 17 days. Animals were exposed to the FST at 24 h after the last drug administration. This procedure consisted of a pretest (day 18) and test (day 19), as described below. SAC at 120 mg/kg showed the best effect in the FST and these same mice receiving this dose, as well as of imipramine and control groups, used for testing behavioral responses, were analyzed for biochemical assays. Animals were added if necessary to complete the study.

To analyze spontaneous locomotor activity, mice were included in three treatment groups without FST exposure: vehicle (ip; *n* = 11), imipramine (15 mg/kg, ip; *n* = 13), and SAC (120 mg/kg, ip; *n* = 15).

Experimental procedures for oxidative stress assays (ROS levels, O_2_^•−^ production, LPx, and antioxidant enzymes activities) were performed after the FST. Mice were allocated to four experimental groups as previously described. Then, thirty min after FST (day 19), mice were euthanized by decapitation to obtain the prefrontal cortex (PFC), midbrain (MB), and hippocampus (HP).

In particular, ROS levels and O_2_^•−^ production were analyzed on the same animals. The samples from each brain region were assayed in 27 mice (ROS, *n* = 4–7 animals per group; O_2_^•−^, *n* = 4–5 animals per group). LPx was performed on 37 mice (*n* = 8–10 animals per group).

The antioxidant enzymes’ activities (Mn-SOD, Cu,Zn-SOD, GPx, and GR) were assayed on the same mice from animals used to test the antidepressant-like effects of SAC (120 mg/kg), imipramine and vehicle groups (*n* = 33). Animals used for this assay were for Mn-SOD (*n* = 6–8 mice per group), Cu,Zn-SOD (*n* = 6–8 mice per group), GPx (*n* = 7–9 mice per group), and GR (*n* = 7–9 mice per group). Aliquots of the tissue homogenate of each animal were measured, where samples were available for each of the measurements.

### 2.4. FST

Antidepressant-like responses were estimated in the FST, with minor modifications as described previously [22]. Mice were individually placed in a glass cylinder (12 cm in diameter, 30 cm high), and filled with tap water (25 ± 1 °C) at the height of 15 cm.

The test consisted of two exposures to the cylinder. On the first day (pre-test, 18 d), the mice were placed individually for 15 min in the glass cylinder. After 24 h (test, 19 d), the exposure was repeated but swim duration was reduced to a 6 min period. The total duration of passive floating (immobility behavior) was measured during this period. Immobility was established as floating passively in an upright position in the water, with only small movements made necessary to keep the head above the water surface.

### 2.5. Spontaneous Locomotor Activity

In order to investigate whether the immobility time in the FST is involved in the alterations of locomotor activity, we analyzed spontaneous locomotor activity using a method previously described [23]. Mice were administered with SAC (120 mg/kg, ip), imipramine (15 mg/kg, ip), or saline daily for 17 days, and 60 min after the last administration the experimental procedure was performed. Locomotor activity was measured on day 17 by using an activity meter Opto-Varimex minor (Columbus Instruments, Int., Columbus, OH, USA). The locomotion associated with ambulation was defined as the total distance traveled in 10 min.

### 2.6. ROS Determination

ROS levels were measured in the homogenates of MB, PFC, and HP by a modified spectrophotometric method [24,25] based on the oxidation of DCFH-DA to DCF. The homogenates were combined with 50 mM sodium phosphate buffer pH 7.4 and 5 µM DCFH-DA. DCF formation was followed for 30 min at 500 nm in a spectrophotometer (Beckman Coulter Inc., Brea, CA, USA). The results were indicated as the change in optical density (O.D.)/mg protein.

### 2.7. O_2_^•−^ Production Assay

O_2_^•−^ is an important ROS and was measured by a previously described method [24]. O_2_^•−^ in brain tissue was evaluated through oxidation of DHE to ethidium (Eth). Homogenates were incubated with DHE (0.2 mM), salmon testes DNA (10 mg/mL), and 50 mM sodium phosphate buffer pH 7.4. Eth-DNA fluorescence was measured at 480 nm excitation and 610 nm emission in a Synergy HT multimode microplate reader (Biotek Instruments, Inc., Winooski, VT, USA). The results were expressed as fluorescence units/mg protein [25].

### 2.8. LPx Analysis

LPx, a widely oxidative stress marker that reflects lipid oxidation, was evaluated through thiobarbituric acid-reactive substances (TBA-RS) production, as reported previously by us [15]. This method mainly measures malondialdehyde (MDA) levels, a compound that results from the decomposition of polyunsaturated fatty acids. Brain regions were homogenized in 2 mL of a solution containing 50 mM phosphate buffer (pH 7.0), 15 mM NaCl, and 145 mM KCl.

One milliliter of homogenate was stirred with 2 mL of the TBA reagent (containing 26 mM TBA, 211 mM HCl and 6.6% TCA), and the mix was boiled for 30 min, centrifuged at 2000× *g*, 4 °C for 10 min. The absorbance at 532 nm was read on an Epoch spectrophotometer (BioTek Instruments, Inc., Winooski, VT, USA). The results were expressed as nmoles of TBA-RS/mg protein. All samples were assayed in duplicate.

### 2.9. Antioxidant Enzymes

We analyzed the antioxidant system measuring the antioxidant enzymes activities, including Mn-SOD, Cu,Zn-SOD, GPx, and GR in brain homogenate tissue (PFC, MB, and HP).

#### 2.9.1. SOD Activity

The assay is based on the inhibition of O_2_^•−^-induced reduction of NBT to formazan by SOD as previously described [26]. For total SOD activity, 330 µL of tissue homogenate was mixed with 2.45 mL of the reaction mixture (122 µM xanthine, 30.6 µM NBT, 122 µM EDTA, 49 mM sodium carbonate, and 0.006% BSA). The reaction started with the addition of 50 µL of xanthine oxidase (2.8 U/L). After 30 min at 27 °C, reactions were stopped by addition of 660 µL of 0.8 mM CuCl_2_, and absorbance at 560 nm was read. By using 50 mM DDC, Cu,Zn-SOD was inhibited, and then Cu,Zn-was calculated from the difference between total SOD and Mn-SOD activities. One unit is the amount of SOD in total that inhibits the rate of formazan formation by 50%. Samples were measured in duplicate, and data were expressed as units/mg protein.

#### 2.9.2. GPx Activity

The principle of this assay is that GPx catalyzes the reduction of H_2_O_2_, oxidizing GSH to form GSSG, which is recycled to its reduced form by GR and NADPH [8]. Tissue homogenate was added to the reaction mixture (50 mM potassium phosphate buffer (pH 7.0), 1 mM EDTA, 1 mM sodium azide, 0.2 mM NADPH, 1 U/mL of GR, and 1 mM GSH). It was then allowed to incubate for 5 min at room temperature before initiation of the reaction by the addition of 0.1 mL of 1.25 mM H_2_O_2_. Optical density was read at 340 nm on a Beckman DU-640 spectrophotometer (Beckman Coulter, Inc., Brea, CA, USA) every 60 s for 3 min. The activity was determined from the slope of these lines (µmoles of NADPH oxidized per min) using a molar extinction coefficient of NADPH (6.22 × 10^−3^ M^−1^ cm^−1^). Data are reported as units/mg protein.

#### 2.9.3. GR Activity

The assay is based on the reduction of GSSG by NADPH in the presence of GR [8]. Fifty microliters of brain homogenate were added to 950 µL of reaction mixture (0.10 M sodium phosphate buffer (pH 7.6), 1 mM GSSG, 0.1 mM NADPH, and 0.5 mM EDTA), and the absorbance was measured at 340 nm, every 60 s for 3 min. The activity was calculated from the slope of these lines (µmoles of NADPH oxidized per min) using a molar extinction coefficient of NADPH (6.22 × 10^−3^ M^−1^ cm^−1^). Data are reported as units/mg protein.

### 2.10. Statistical Analysis

One-way analyses of variance (ANOVA) and post hoc Duncan’s tests (SPSS Statistics 20 software; SPSS Inc., Chicago, IL, USA) were used to compare data. Values of *p* < 0.05 were considered to be statistically significant.

## 3. Results

### 3.1. SAC Exhibits an Antidepressant-Like Effect in the FST

**Figure 2:**
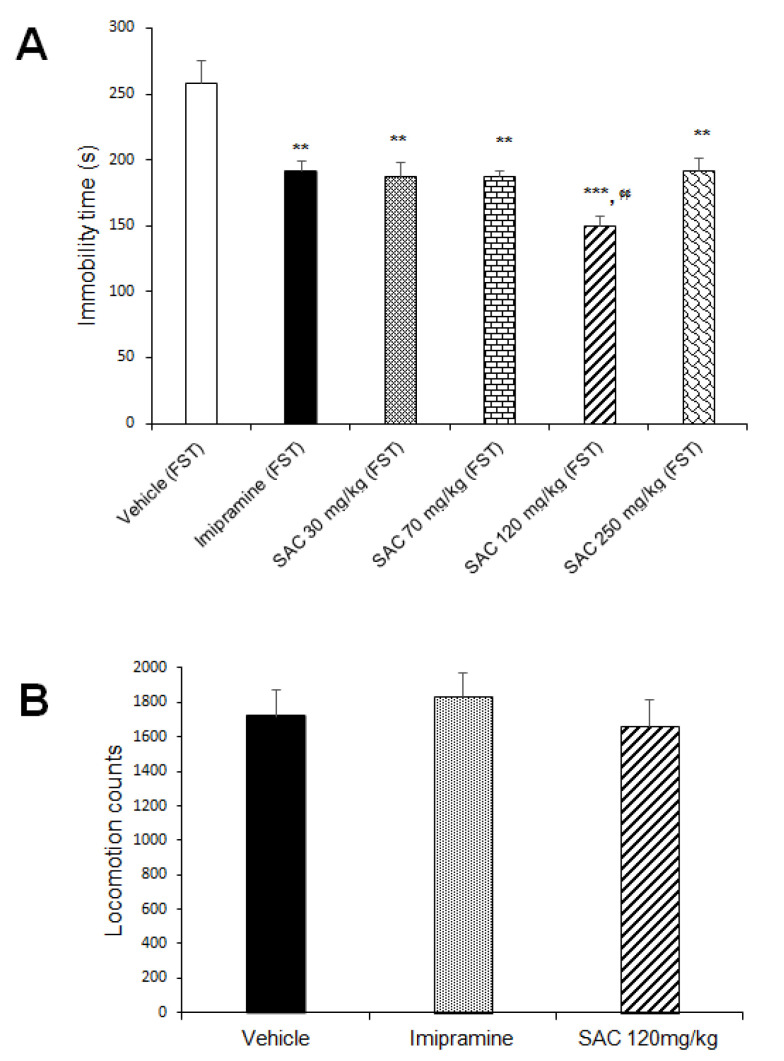
SAC produced an antidepressant-like response in the FST (**A**) and did not alter locomotor activity (**B**). Before FST, vehicle, imipramine (15 mg/kg), or SAC (30, 70, 120, or 250 mg/kg) were administered to mice daily for 17 days. Immobility time (in seconds) and locomotion scores are expressed as mean ± SEM, with 9–15 mice per group for antidepressant-like response and 11–15 mice per group for locomotor activity. The one-way ANOVA followed by post hoc Duncan’s test was used to analyze differences among groups. (**) Statistically different from the vehicle-FST group (control group, *p* < 0.01, Duncan´s test; (***) Statistically different from the vehicle-FST group (control group), *p* < 0.001, Duncan´s test; (¢¢) statistically different from imipramine (FST) group, *p* < 0.01, Duncan´s test. FST = forced swimming test; SAC = S-allyl cysteine.

The one-way ANOVA showed a statistically significant response for SAC and imipramine (classical antidepressant) on immobility scores (F (5, 69) = 17.614, *p* = 0.000). Both significantly reduced the duration of immobility in the FST (Figure 2A), suggesting that SAC produces an antidepressant-like response. This effect was statistically significant with reduction of 30%, 31%, 44%, and 29% for 30, 70, 120, and 250 mg/kg of SAC, respectively, when compared to the vehicle-treated control group (Duncan post-hoc *p* < 0.001). SAC at 120 mg/kg was used to analyze ROS levels, O_2_^•−^ production, LPx, spontaneous locomotor activity, and the activity of different antioxidant enzymes because it was the most effective dose.

### 3.2. Antidepressant-Like Response to SAC is Not Related to Altered Spontaneous Locomotor Activity

Data obtained in the spontaneous locomotor activity test were analyzed in the different groups (Figure 2B). SAC (120 mg/kg) and imipramine did not alter locomotor activity as compared to the vehicle-treated control group (F (2, 36) = 0.404, *p* = 0.671). This suggests that SAC exerts a selective antidepressant-like response since spontaneous locomotor activity was not affected.

### 3.3. Effect of SAC on ROS Levels in Mice Exposed to FST

**Figure 3:**
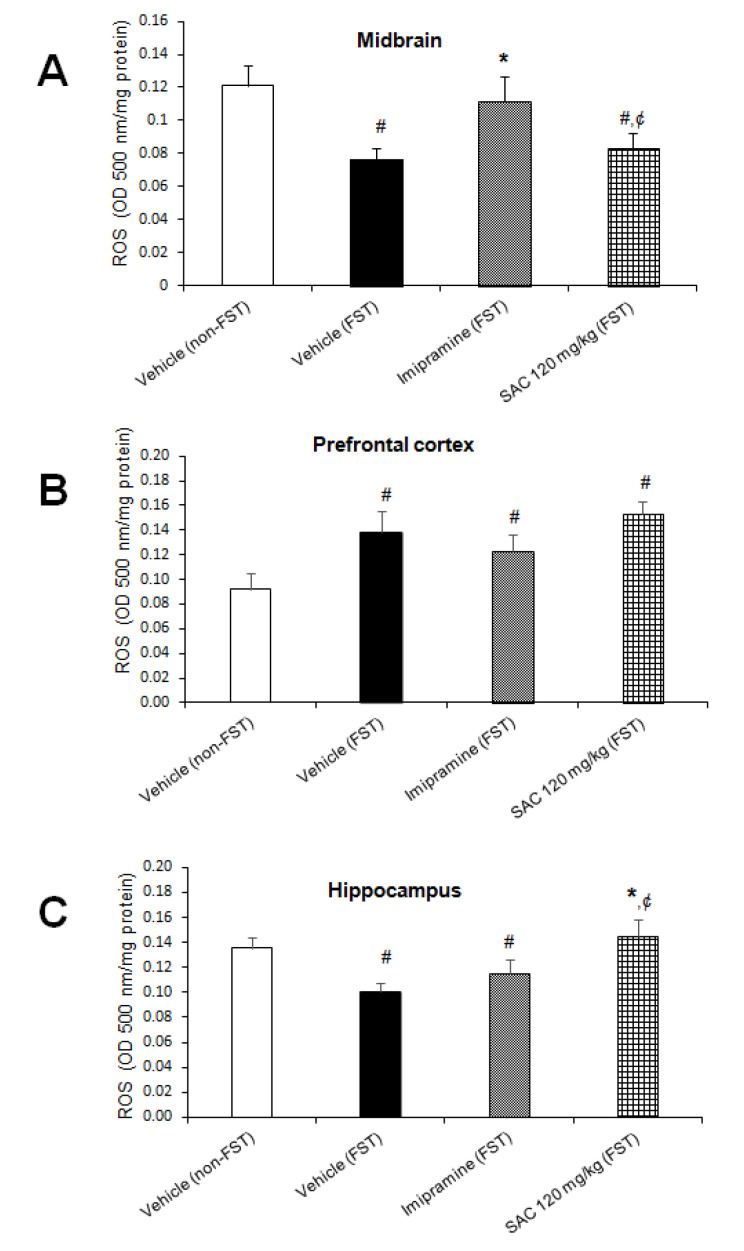
Effect of SAC on ROS levels in several brain regions of animals subjected to FST. The vehicle (non-FST), vehicle (FST), imipramine (FST) or SAC (120 mg/kg) were the treatment groups. SAC did not reduce the ROS amount in the midbrain (**A**), prefrontal cortex (**B**), or hippocampus (**C**) in the FST vs. vehicle-FST group. Results are expressed as mean ± SEM of 4–7 animals per group. The one-way ANOVA followed by post hoc Duncan’s test was used to analyze differences among the groups. (#) statistically different from vehicle non-FST group, *p* < 0.05, Duncan’s test; (*) statistically different from vehicle-FST group, *p* < 0.05; (¢) statistically different from imipramine (FST) group, *p* < 0.05, Duncan´s test. FST = forced swimming test; SAC = S-allyl cysteine; ROS = reactive oxygen species.

The amount of ROS in different brain regions is shown in Figure 3. The one-way ANOVA exhibited statistically significant differences among the groups (MB: F (3, 23) = 3.75, *p* = 0.026, Figure 3A; PFC: F (3, 15) = 3.214, *p* = 0.05, Figure 3B; HP: F (3, 20) = 3.71; *p* = 0.029, Figure 3C). Post hoc multiple comparison tests (Duncan post hoc *p* < 0.05) showed that the vehicle-FST group reduced ROS levels in MB as compared to the non-swimming group (36%). However, ROS levels were increased in the same brain region in the imipramine-FST group vs. the FST-group (46%), SAC (120 mg/kg) administered to FST group showed a statistically significant reduction in ROS levels in MB compared to the imipramine-FST group (25%) and non-FST group (31%).

We have shown that FST was able to increase the ROS levels in PFC as compared to the non-FST group (50%, Figure 3B). SAC (120 mg/kg) and imipramine administration in animals exposed to FST did not change the enhancement of ROS. In particular, FST group produced a significant reduction in ROS formation in HP (25%, Figure 3C), but the SAC-FST group increased ROS formation as compared to the vehicle-FST group (44%, Figure 3C) and to the imipramine-FST group (26%, Figure 3C).

### 3.4. Effect of SAC on O_2_^•−^ Production in Mice Exposed to FST

**Figure 4:**
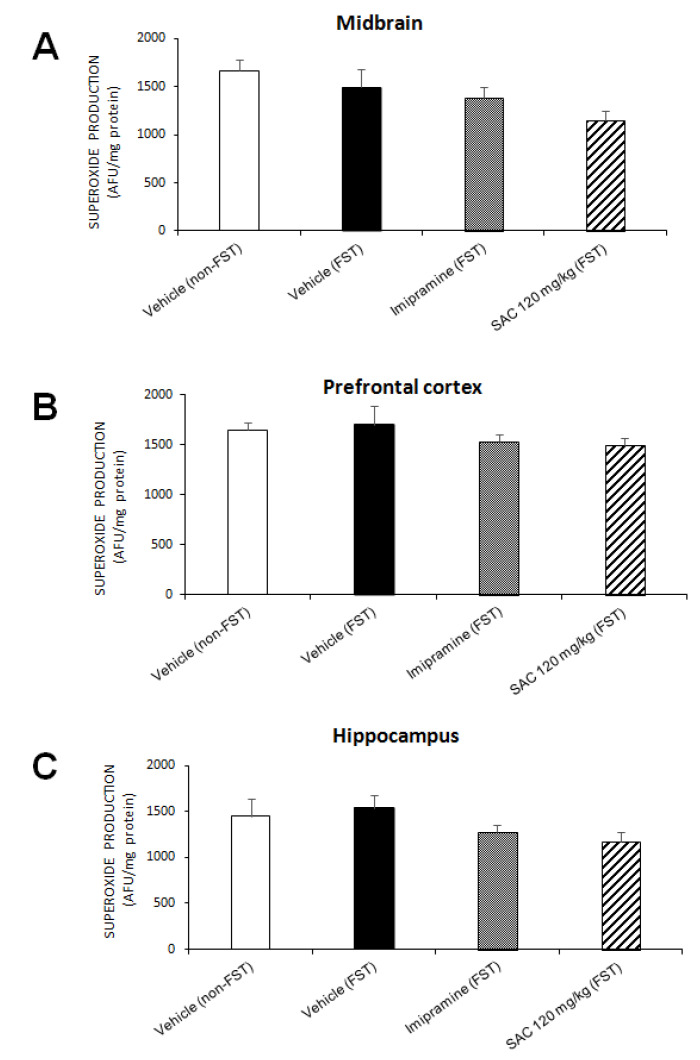
Effect of SAC on O_2_^•−^ production in several brain regions in mice subjected to FST. The vehicle (non-FST), vehicle (FST), imipramine (FST) or SAC (120 mg/kg) were the treatment groups. SAC did not reduce O_2_^•−^ production in the midbrain (**A**), prefrontal cortex (**B**), or hippocampus (**C**) in the FST vs. vehicle-FST group. Results are expressed as mean ± SEM of 4–5 animals per group. The one-way ANOVA was used to analyze results. FST = forced swimming test; SAC = S-allyl cysteine; AFU = arbitrary fluorescence units; O_2_^•−^ = superoxide radical.

O_2_^•−^ is an important ROS that can cause oxidative damage. No changes were found in MB (F (3, 13) = 2.198, *p* = 0.137; Figure 4A), PFC (F (3, 16) = 0.699, *p* = 0.566; Figure 4B), and HP (F (3, 13) = 1.645, *p* = 0.027; Figure 4C) in the different treatment groups. However, SAC (120 mg/kg) treatment showed a trend to reduce O_2_^•−^ production in MB and HP.

### 3.5. Antidepressant-Like Behavior exerted by SAC Is Related with the Decrease in LPx in HP in Mice Exposed to FST

**Figure 5:**
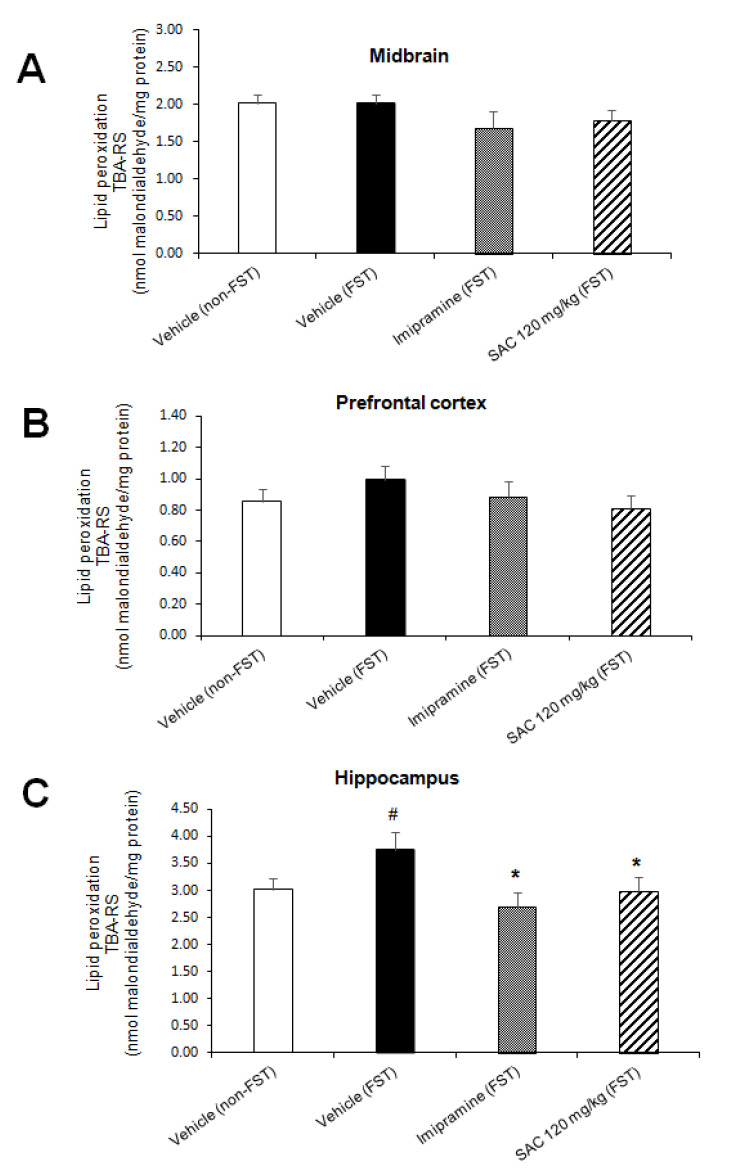
The antidepressant-like behavior of SAC is associated with a reduction in lipid peroxidation in the hippocampus of animals subjected to FST. The vehicle (non-FST or FST), imipramine (FST) or SAC (120 mg/kg) were the treatment groups. The thiobarbituric acid-reactive substances (TBA-RS) assay was used to measure lipid peroxidation in the midbrain (**A**), prefrontal cortex (**B**), and hippocampus (**C**). Results are expressed as mean ± SEM of 8–10 animals per group. The one-way ANOVA followed by post hoc Duncan’s tests was used to analyze the differences among groups. (#) Statistically different from vehicle non-FST group, *p* < 0.05, Duncan’s test; (*) statistically different from vehicle (FST) group, *p* < 0.05, Duncan´s test. FST = forced swimming test; SAC = S-allyl cysteine.

To analyze whether the antidepressant-like effect of SAC (120 mg/kg) was associated in preventing damage caused by oxidative stress, the TBA-RS assay provided an index of LPx (Figure 5).

There was a statistically significant difference in LPx in HP among the groups (F (3, 31) = 2.919, *p* = 0.05; Figure 5C). Post hoc multiple comparisons (Duncan post-hoc *p* < 0.05) showed a significant increase in LPx after FST as compared to the non-FST group (24%). Imipramine administration reduced LPx after FST vs. the vehicle-FST group (28%). We demonstrated that SAC (120 mg/kg) administered to mice exposed to FST produces a significant reduction in LPx of HP vs. the vehicle-FST group (20%, Figure 5C). No changes were found in MB (F (3, 31) = 1, *p* = 0.406; Figure 5A) and PFC (F (3, 29) = 0.805; *p* = 0.501; Figure 5B) in different treatment groups.

### 3.6. Effect of SAC on Antioxidant Enzymes Activities in Mice Subjected to FST

To investigate whether SAC (120 mg/kg) produces a protective effect via enzymatic antioxidant mechanisms in mice exposed to FST, we analyzed Mn-SOD, Cu,Zn-SOD, GPx, and GR activities.

**Figure 6:**
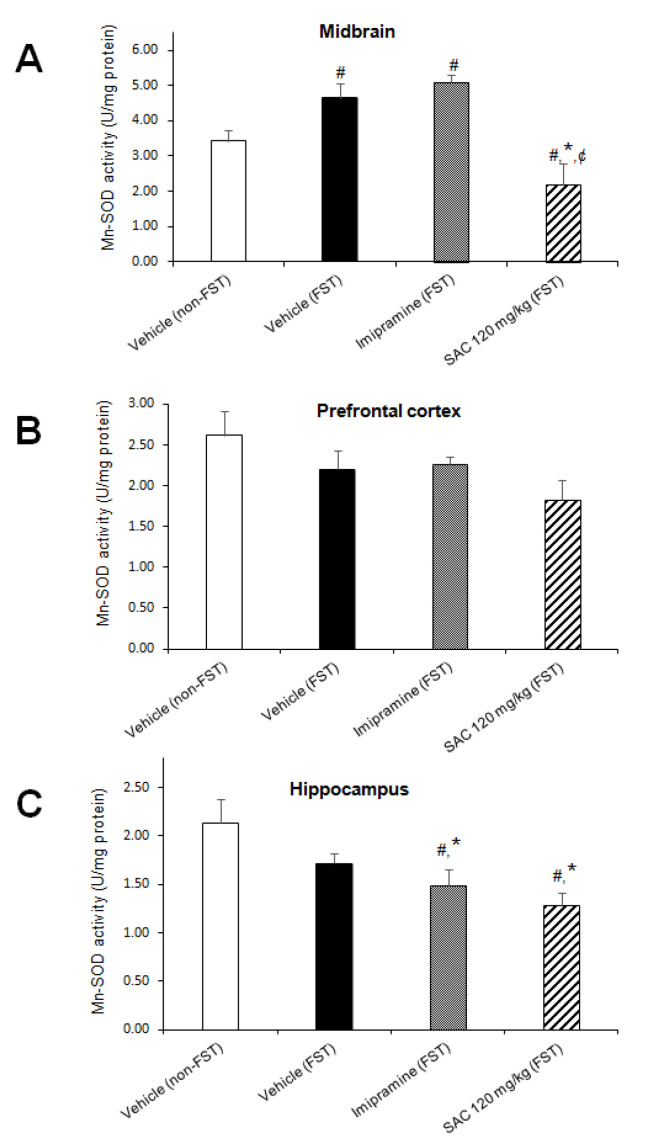
The antidepressant-like behavior exhibited by SAC (120 mg/kg) in the FST is related with downregulation of Mn-SOD activity in midbrain and hippocampus. The vehicle (non-FST or FST), imipramine (FST) or SAC (120 mg/kg) were the treatment groups. Mn-SOD was analyzed in the midbrain (**A**), prefrontal cortex (**B**), and hippocampus (**C**). Results are expressed as mean ± SEM of 6–8 animals per group. The one-way ANOVA followed by post hoc Duncan’s tests was used to analyze the differences among groups. (#) Statistically different from the non-FST group, *p* < 0.05, Duncan’s test; (*) statistically different from the vehicle-FST group, *p* < 0.05, Duncan’s test; (¢) statistically different from the imipramine-FST group, *p* < 0.05, Duncan’ test. Mn-SOD = manganese-superoxide dismutase; FST = forced swimming test; SAC = S-allyl cysteine.

The one-way ANOVA showed significant differences in Mn-SOD activity among groups in MB (F (3, 23) = 9.696, *p* = 0.000, Figure 6A), and HP (F (3, 25) = 4.662; *p* = 0.010; Figure 6C). Post hoc multiple comparisons (Duncan post-hoc *p* < 0.05) showed that FST enhanced Mn-SOD activity in MB as compared to the non-swimming group (36%, Figure 6A). However, FST did not change Mn-SOD activity in the HP vs. the non-FST group.

On the other hand, FST did not alter Mn-SOD activity in the imipramine group in MB when compared to the control group (vehicle-FST). However, a reduction in Mn-SOD activity in HP was shown by the imipramine group exposed to FST (13%, Figure 6C) vs. the FST group. SAC (120 mg/kg) reduced Mn-SOD activity in MB (53%; Figure 6A), and HP (25%, Figure 6C) after FST compared to the vehicle-FST group. It is suggested that an early mechanism induced by SAC is acting as an O_2_^•−^ scavenger that prevents enhancement of Mn-SOD activity. SAC (120 mg/kg) reduced Mn-SOD activity in MB after FST compared to the imipramine-FST group (62%, Figure 6A).

No changes were found in PFC in the different treatment groups (F (3, 26) = 2.076; *p* = 0.128; Figure 6B).

**Figure 7:**
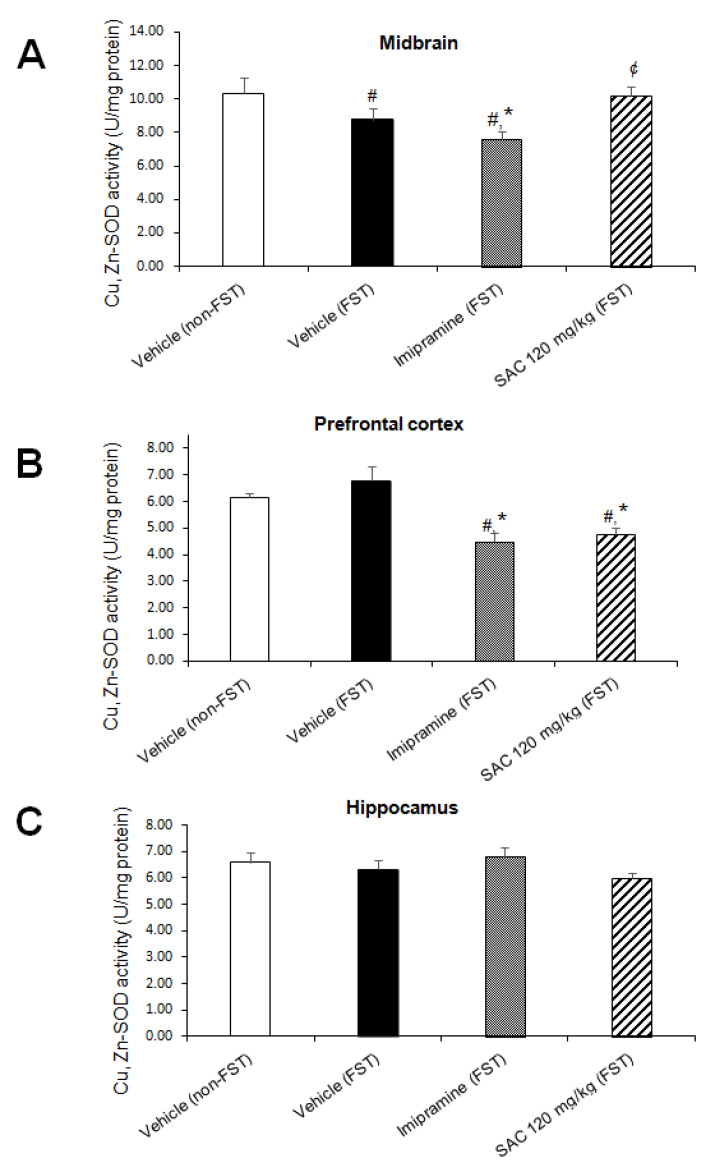
The antidepressant-like response of SAC (120 mg/kg) in the FST is related with downregulation of Cu,Zn-SOD activity in prefrontal cortex. The vehicle (non-FST or FST), imipramine (FST) or SAC (FST) were the treatment groups. Cu,Zn-SOD was analyzed in the midbrain (**A**), prefrontal cortex (**B**), and hippocampus (**C**). Results are expressed as mean ± SEM of 6–8 animals per group. The one-way ANOVA followed by post hoc Duncan’s tests was used to analyze the differences among groups. (#) Statistically different from the non-FST group, *p* < 0.05, Duncan’s test; (*) statistically different from the vehicle-FST group, *p* < 0.05, Duncan’s test; (¢) statistically different from the imipramine-FST group, *p* < 0.05, Duncan’s test. Cu,Zn-SOD = copper, zinc-superoxide dismutase; FST = forced swimming test; SAC = S-allyl cysteine.

There was a statistically significant difference in Cu,Zn-SOD activity among the groups in MB (F (3, 22) = 4.010, *p* = 0.02; Figure 7A) and PFC (F (3, 25) = 9.784, *p* = 0.000; Figure 7B), but not in HP. Post hoc multiple comparisons (Duncan post hoc *p* < 0.05) showed a significant reduction in MB (15%, Figure 7A) after FST when compared to the non-swimming group (non-FST). Imipramine administration reduced Cu,Zn-SOD activity in MB (13%, Figure 7A) as well as in PFC (34%, Figure 7B) after FST (imipramine-FST vs. vehicle-FST). However, SAC (120 mg/kg) enhanced Cu,Zn-SOD activity in MB (34%, Figure 7A) as compared to the imipramine-FST group and decreased it in PFC after FST compared to the vehicle-FST group (29%, Figure 7B). This reduction in PFC suggests that SAC exerts a previous mechanism as a free radical scavenger which prevents the enhancement of Cu,Zn-SOD in this brain region. No changes were found in HP in the different treatment groups (F (3, 28) = 1.151; *p* = 0.346; Figure 7C).

**Figure 8:**
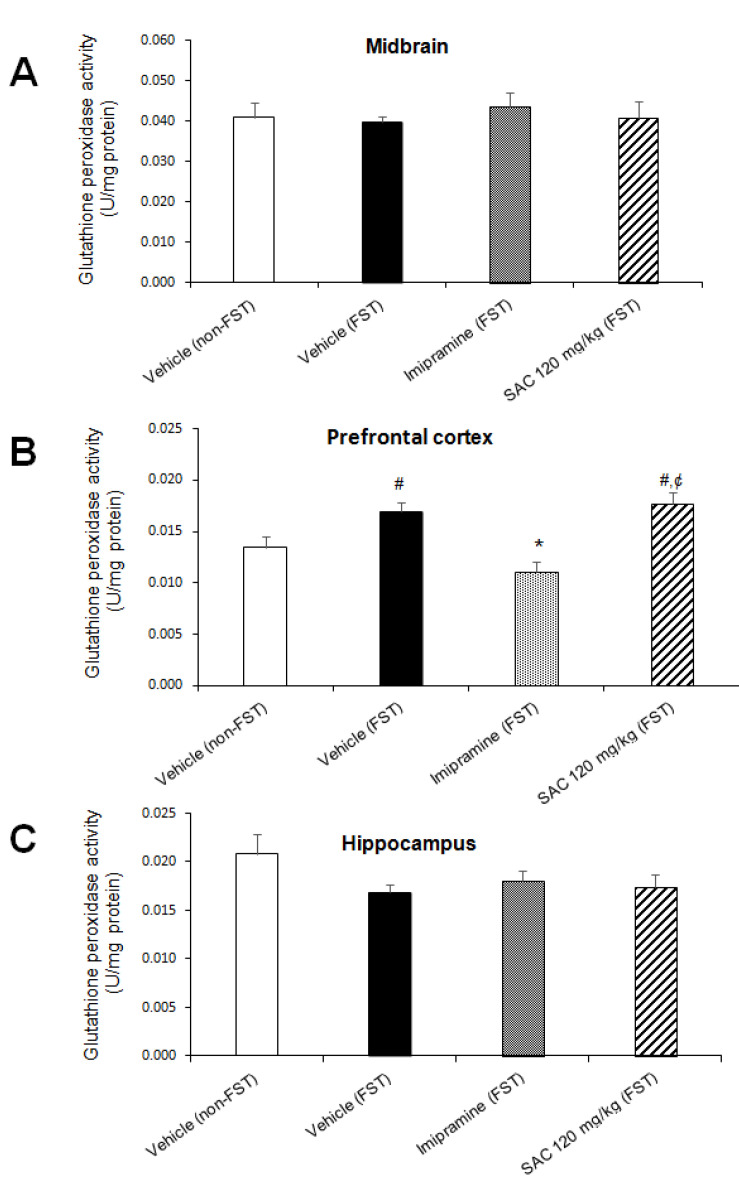
Effect of SAC on GPx activity in different brain regions in mice subjected to FST. The vehicle (non-FST or FST), imipramine (FST) or SAC (120 mg/kg) were the treatment groups. SAC did not change GPx activity in the midbrain (**A**), prefrontal cortex (**B**), or hippocampus (**C**) in FST vs. vehicle-FST group. Results are expressed as mean ± SEM of 7–9 animals per group. The one-way ANOVA followed by post hoc Duncan’s tests was used to analyze the differences among groups. (#) statistically different from vehicle non-FST group, *p* < 0.05, Duncan’s test; (*) statistically different from vehicle-FST group, *p* < 0.05, Duncan’s test; (¢) statistically different from imipramine (FST) group, *p* < 0.05, Duncan’s test. FST = forced swimming test; SAC = S-allyl cysteine; GPx = glutathione peroxidase.

We found that GPx activity was significantly different among groups in PFC (F (3, 26 = 9.744, *p* = 0.000; Figure 8B). Post hoc multiple comparisons (Duncan post hoc *p* < 0.05) showed a significant enhancement after FST as compared to the non-FST group (25%). Imipramine reduced GPx activity after FST compared to the control group (vehicle-FST, 34%). However, SAC (120 mg/kg) treatment enhanced GPx activity in mice exposed to FST versus the imipramine-FST group (59%, Figure 8B). No differences were found in MB (F (3, 29) = 0.247; *p* < 0.862; Figure 8A) and HP (F (3, 28) = 1.603; *p* = 0.211; Figure 8C). 

**Figure 9:**
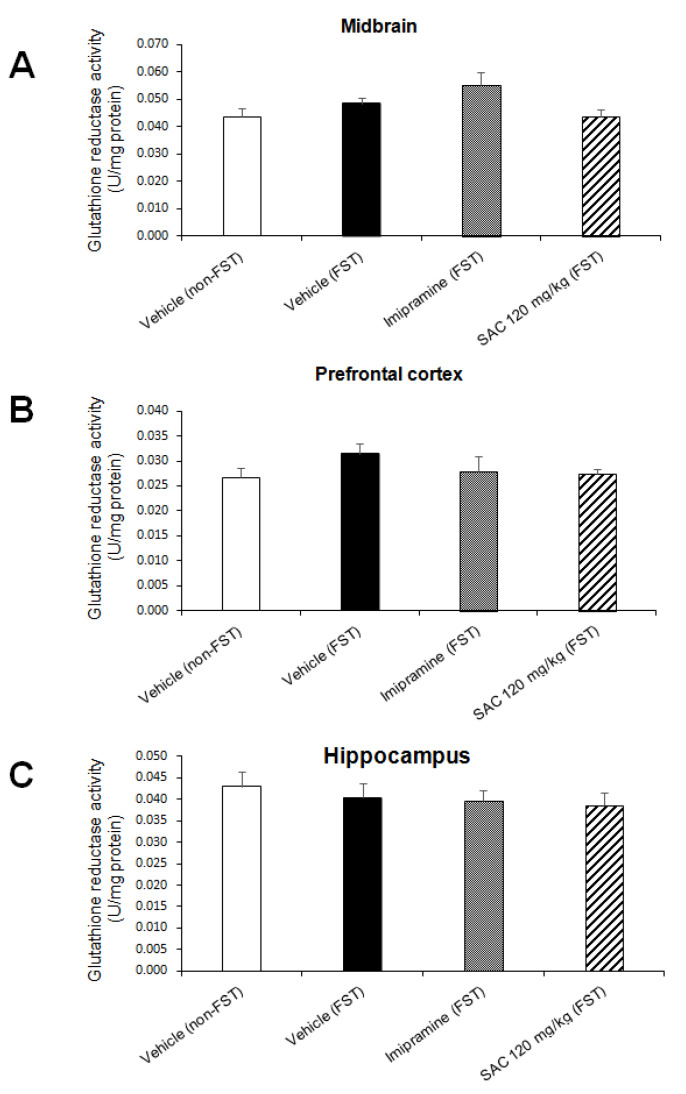
Effect of SAC on GR activity in different brain regions in mice exposed to FST. The vehicle (non-FST or FST), imipramine (FST) or SAC (120 mg/kg) were the treatment groups. SAC did not change GR activity in the midbrain (**A**), prefrontal cortex (**B**) or hippocampus (**C**) in FST vs. vehicle-FST group. Results are expressed as mean ± SEM of 7–9 animals per group. Results were analyzed using one-way ANOVA. FST = forced swimming test; SAC = S-allyl cysteine; GR = glutathione reductase.

No changes were found in GR activity (Figure 9) in MB (F (3, 28) = 2.688; *p* = 0.066, PFC (F (3, 25) = 0.942; *p* = 0.435) and HP (F (3, 29) = 0.374; *p* = 0.773).

## 4. Discussion

Treatment for depression using antidepressant drugs results in remission, but with severe adverse effects and low response rates. The new therapies from natural resources have been used in psychiatric treatment due to greater patient compliance and mild side effects [27]. In particular, SAC, an active compound of aged garlic extract, is a potent antioxidant agent [14], with neuroprotective effects against oxidative damage in diverse animal models [14,15,16]. In this sense, it has been suggested that oxidative stress plays an essential role in depression [5,28], as well as in animal models for depression-like behavior [8,19,20]. Therefore, antioxidant compounds could have an antidepressant effect, which is supported by preclinical studies [29]. The current study introduces the antidepressant-like effect of SAC, accompanied, mainly, by a reduction in hippocampal oxidative damage induced by the FST model.

This research reports that the antidepressant-like effect of SAC was present at the four doses tested. The highest protection was observed at the 120 mg/kg dose, which produced a significant decrease (44%) in immobility time in the FST, with a better profile than the classical antidepressant imipramine (29%). We analyzed spontaneous locomotor activity to avoid false positives in the FST, to exclude the possibility that the reduction in immobility time produced by SAC may be associated with alterations in locomotor activity. Thus, we found that SAC treatment at 120 mg/kg did not affect this behavior, suggesting that this compound exerts a selective antidepressant-like response.

Additionally, FST is an animal test that can induce oxidative stress [8,19]. Therefore, we explored whether antidepressant-like effects of SAC are related to its anti-oxidative role in this model. For this purpose, we analyzed different oxidative stress markers (ROS levels, O_2_^•−^ production, LPx, and antioxidant enzymes activities) in three different brain regions; the MB, PFC, and HP.

These brain regions are related to symptoms commonly presented in depressed patients [30] and form part of two circuits. The first is the mesocortical pathway, which originates from ventral tegmental area (VTA), localized in the MB, and projects to the PFC and anterior cingulate cortex. The mesolimbic pathway also projects from VTA to different brain regions including HP. Our results show that the SAC effect in the FST produced a reduction in some oxidative stress markers in the brain regions analyzed. No changes were found in O_2_^•−^ production and LPx, nor GPx, GR, and Cu,Zn-SOD activities in MB after SAC administration in animals exposed to FST vs. the vehicle-FST group, although SAC treatment showed a trend to reduce O_2_^•−^ production. Nevertheless, Mn-SOD activity in MB was reduced significantly in the SAC group exposed to FST. It is suggested that an early mechanism induced by SAC during the first day is acting as an O_2_^•−^ scavenger that prevents enhancement of Mn-SOD activity on the second day. Mn-SOD facilitates dismutation of O_2_^•−^ that produces H_2_O_2_ in mitochondria, and it is removed by catalase and GPx enzymes preventing the formation of hydroxyl radicals [31]. However, the reduction in Mn-SOD activity by SAC did not change, indirectly suggesting no generation of H_2_O_2_, and therefore no changes in GPx activity.

Another important region is the PFC that participates in the temporal organization of behavior [32], and supports cognitive abilities that are required to organize behavior in time and context through connections with the limbic (including HP), cognitive, sensory, and motor regions following stimulation. In our study, the analysis of oxidative markers in PFC showed no changes in ROS levels, O_2_^•−^ production, LPx, Mn-SOD, GPx, or GR activities after SAC administration in the FST model. However, SAC significantly decreased Cu,Zn-SOD activity in PFC in mice exposed to FST. This also suggests that SAC prevented oxidative stress, in part, due to O_2_^•−^ scavenging effect, since Cu,Zn-SOD activity was reduced in PFC. This could be related to a previous mechanism as a free radical scavenger due to SAC on the first day of the FST which prevents enhancement of Cu,Zn-SOD on the second day of the test.

The HP plays an important role in the adjustment of learning, memory and emotion regulation [33] of contextual information of the environment. The oxidative stress assays in HP showed no differences in O_2_^•−^ production, nor Cu,Zn-SOD, GPx, and GR activities after SAC administration in the FST model. However, SAC treatment showed a trend to reduce O_2_^•−^ production. Nonetheless, SAC administration produced a significant reduction in LPx and Mn-SOD activity after FST compared to the control group (vehicle-FST). This suggests that SAC prevented LPx related to scavenging O_2_^•−^ on the first day of FST, since mitochondrial antioxidant Mn-SOD activity was reduced on the second day of FST.

These results support the role of oxidative stress in depression [2]. The enhancement of MDA levels, an index of LPx, has been reported in major depression in samples of serum [34], as well as peripheral blood [34]. Moreover, treatment with antidepressants reduced MDA levels [35]. We found that FST elevated LPx in HP as previously reported [8], and SAC prevented this effect in a similar manner to imipramine. It is also well known that SAC prevents LPx [14], which supports our findings. In addition, a positive correlation was found between increased SOD serum levels and the severity of depressive symptoms [36]. We showed a reduction in Mn-SOD activity in the SAC-FST group compared to the FST group in MB and HP, as well as a reduction in Cu,Zn-SOD activity in the SAC-FST group compared to the FST group in PFC.

In this context, it is well-known that SAC exerts an antioxidant action and scavenges O_2_^•−^ [14]. Additionally, SAC has a neuroprotective effect against oxidative damage in the brain in diverse animal models of neurological/neurodegenerative diseases [15,16,37]. Therefore, it is suggested that an early mechanism induced by SAC during the first day of FST is to act as an O_2_^•−^ scavenger, and preventing the enhancement of Mn-SOD activity in MB and HP, which is indicated by a downregulation of Mn-SOD activity on the second day of the FST where we found an antidepressant-like effect. The same pattern was found but with a reduction in Cu,Zn-SOD activity in PFC. Our findings suggest that SAC produced activation of an endogenous antioxidative mechanism with the first line of defense related to mitochondrial antioxidant Mn-SOD activity in HP since two oxidative stress markers were reduced in this brain region.

SOD is a very important enzyme in reducing oxidative stress. It has been reported that Mn-SOD is abundant in neurons, and Cu,Zn-SOD is enriched in astrocytes with lower levels in neurons [38]. These antioxidant enzymes have different distribution in brain regions [39,40]. However, neurons are highly vulnerable to a reduction in Mn-SOD as compared to Cu,Zn-SOD. In addition, SOD activity results in H_2_O_2_ production, but if catalase or GPx do not remove it, then the SOD overexpression can exacerbate brain damage. In addition, neurons are unable to enhance the expression of GPx, even with an increase in free radical production [41]. Therefore, neurons may have a limited capacity for ROS detoxification, which can interact with cellular lipids, proteins, and DNA, leading to cellular dysfunction and sometimes cell death.

The present study showed that SAC administration in mice exposed to FST resulted in brain region-specific changes, with the main changes being in the HP. This can be related to the modulation of the response in each region, attributed to compensatory mechanisms to counteract the possible detrimental effects associated with oxidative stress, as well as different properties of the stressor with multiple physiological effects, distinct cell composition for each brain region [42], and different metabolic rate to process information per brain region [43]. Additionally, the role of biological (strain, age, body, weight, gender, individual differences between animals), environmental, and physical factors (light, noise, food manipulations, handling, enriched environment, schedule, routes of treatment, time-course of analysis) should be considered.

In this regard, we reported differences on the second day of the FST, although several changes may occur during the first day of FST, which was not analyzed. It is important to note that different molecular mechanisms are activated during the first hours of exposure; for example, enhancement of hippocampal RNA expression of glucocorticoid-responsive genes [44], increase in hippocampal mineralocorticoid receptors [45], and epigenetic changes related to gene transcription [46]. Epigenetic processes may play an important role in stress-related mental disorders such as depression [47]. Further studies need to be performed during the first day of FST to explore the early molecular mechanisms leading to the SAC antidepressant-like effect on the second day in the different brain regions. It is also important to note that SAC is widely used, has notable clinical safety evidence, and crosses the blood–brain barrier [14], and is therefore a candidate for further investigation, especially for the treatment of depression.

## 5. Conclusions

We showed that SAC has antidepressant-like properties, which could be associated, in part, with its antioxidant role mainly in the HP, and a modulated response in other brain regions. Our results indicate that the antidepressant-like effect of SAC was slightly higher (44%) than the effect observed with a classical antidepressant drug, imipramine (29%), in the FST. However, our findings need to be repeated in other animal tests and models of depression.
